# Playing the long game: A multivariate multilevel non-linear growth curve model of long-term effects in a randomized trial of the Good Behavior Game

**DOI:** 10.1016/j.jsp.2021.08.002

**Published:** 2021-10

**Authors:** Patricio Troncoso, Neil Humphrey

**Affiliations:** a Institute for Social Policy, Housing and Equalities Research (I-SPHERE), Heriot-Watt University, Edinburgh Campus, Scotland EH14 4AS, United Kingdom; b Manchester Institute of Education, The University of Manchester, Oxford Road, Manchester M13 9PL, United Kingdom

**Keywords:** Behavior management, Intervention, Randomized trial, Growth curve, Multilvariate multilevel modeling, Bayes factor

## Abstract

This cluster randomized controlled trial (RCT) examined the impact of the Good Behavior Game (GBG) on children's developmental trajectories of disruptive behavior, concentration problems, and prosocial behavior from middle childhood (ages 6–7 years) to early adolescence (ages 10–11 years). Seventy-seven schools in England were randomly assigned to intervention and control groups. Allocation was balanced by school size and the proportion of children eligible for free school meals. Children (*N* = 3084) ages 6–7 years at baseline were the target cohort. Outcome measures, assessed via the Teacher Observation of Child Adaptation Checklist, were taken prior to randomization (baseline – Time 1) and annually for the next 4 years (Time 2 to Time 5). During the 2-year main trial period (Time 1 to Time 3), teachers of this cohort in intervention schools implemented the GBG, whereas their counterparts in the control group continued their usual practice. A multivariate multilevel non-linear growth curve model indicated that the GBG reduced concentration problems over time. In addition, the model also revealed that the intervention improved prosocial behavior among at-risk children (e.g., those with elevated symptoms of conduct problems at Time 1, *n* = 485). No intervention effects were unequivocally found in relation to disruptive behavior. These findings are discussed in relation to the extant literature, strengths and limitations are noted, and practical and methodological implications are highlighted.

## 1 Introduction

The Good Behavior Game (GBG) is a school-based, universal behavior management intervention implemented by classroom teachers. The core components of the GBG consist of classroom rules, team membership, monitoring behavior, and the use of positive reinforcement. Children work in teams to win the GBG to access agreed-upon rewards. The GBG is played during a normal classroom activity for a specified time period during which the class teacher monitors contraventions to four rules: (a) we will work quietly, or at a noise level appropriate to a given activity; (b) we will be polite to others; (c) we will get out of our seats with permission; and (d) we will follow instructions. Teams with four or fewer rule breaks at the end of the game are deemed the winners and are rewarded. Over time, the game develops in terms of the frequency and duration of gameplay as well as the nature and timing of rewards (Donaldson & Wiskow, 2017). The theoretical bases of the GBG include behaviorism (i.e., contingency management and the replication of rewarded behavior), social learning theory (i.e., learning of appropriate behavior modeled by other team members) and life course/social field theory (i.e., promotion of adaptive processes to enable children to meet social task demands in the classroom; Kellam et al., 2011).

In the decades since its original development (Barrish et al., 1969), 14 randomized controlled trials (RCTs) of the GBG have been conducted of the program being implemented universally during a normal school day. Six of these were conducted in the United States (Dolan et al., 1993; Hansen et al., 2010; Ialongo et al., 2019, Ialongo et al., 1999; Reid et al., 1999; Tolan et al., 2020); two each in the Netherlands (van Lier et al., 2004; Witvliet et al., 2009) and Canada (Dion et al., 2011; Jiang et al., 2018); and one each in Belgium (Leflot et al., 2010), Northern Ireland (O'Keeffe, 2019), Estonia (Streimann et al., 2020), and England (for a review, see Humphrey et al., 2021). The current paper reports on the findings from the RCT conducted in England. A fifteenth RCT based in the United States reports on the impact of the GBG in the context of an afterschool program (Smith et al., 2018). Collectively, these trials have provided robust evidence that the intervention leads to statistically significant changes in a range of salient outcomes, including conduct problems and peer relations, with intent-to-treat (ITT) effect sizes typically in the *g* = 0.10–0.20 range (Smith et al., 2021). This evidence is broadly consistent with intervention effect sizes reported in meta-analyses of a range of universal behavior management approaches (Korpershoek et al., 2016) and school-based preventive interventions more generally (Tanner-Smith, Durlak, & Marx, 2018). Furthermore, this evidence aligns with the view that the behavior of most children is typically not a cause for significant concern (Department for Education, 2012).

In this article we report findings from the first RCT of the GBG in England. The main project report (Humphrey et al., 2018) for this RCT documents the main ITT and subgroup moderator analysis findings in relation to reading scores and behavioral outcomes using point-in-time estimates focusing on the end of the intervention period (noted as Time 3 in *Design* below). In brief, Humphrey et al.'s ITT analysis found no evidence that the GBG improves children's reading or behavior. Furthermore, the subgroup moderator analysis revealed no significant differential gains for children eligible for free school meals or for boys at risk of developing conduct problems.

We build and extend on the above findings in several ways. First, we used growth curve models, as opposed to point-in-time estimates. Second, we examined long-term, as opposed to short-term, intervention effects. Third, these intervention effects were considered at both main and subgroup levels; in terms of the latter, we examined sex and conduct problems risk status, with effects examined separately *and* in combination, as opposed to solely the latter. In doing so, our intended contribution was to extend the knowledge base regarding the scope, specificity, and timing of intervention effects. Put another way, we go beyond *what works* by asking *for which outcomes*? *fFor whom*? and *When*?

Disruptive behaviors (e.g., talking out, getting out of seat, touching others, being disobedient or aggressive) were our primary focus because these behaviors are key proximal outcomes of the GBG (Chan et al., 2012) and are developmentally significant as they are predictive of adult anti-social behavior and related outcomes (e.g., arrest for a violent offence; Hubbard et al., 2006). However, the nature of the intervention also means that reductions in concentration problems (e.g., difficulties in paying attention, staying on task, resisting distractions) and improvements in prosocial behavior (e.g., compliance with rules, demonstrating empathy, social problem-solving) following exposure to the GBG are feasible, and indeed these too are developmentally important. For example, children with early attention difficulties are 40% less likely to graduate from high school (Rabiner et al., 2016).

We focused on intervention effects over the long-term rather than immediately following the conclusion of implementation because, from a theoretical perspective, preventive effects are hypothesized to take time to emerge, especially when a relatively small proportion of the population have, or are at risk of, developing problems in the first place (Hill et al., 2016). Thus, as Greenberg and Abenavoli (2017) noted, complete evaluation of universal interventions requires data collection over extended periods in order for changes among intervention recipients to consolidate, for small but key changes to snowball, and for the members of the control group to exhibit difficulties of the kind that are the focus of prevention efforts. Despite this, long-term follow-up is far from the norm. For example, in Durlak et al. (2011) seminal meta-analysis of universal social and emotional learning interventions, only 15% of studies collected follow-up data at least 6 months after a given intervention ended. Even in cases where long-term follow-up is implemented, studies are limited by a reliance on point-in-time estimates that do not analyze the developmental process of growth, although there are a couple of notable exceptions (e.g., Nix et al., 2016). Because a key purpose of universal interventions is to alter developmental trajectories, it is important that this is reflected in the analytical techniques adopted by researchers (Greenberg & Abenavoli, 2017).

In addition to examining growth over time, it is also important to recognize that the effects of universal interventions may vary across specific strata of the population. In the case of the GBG, there are theoretical and empirical reasons why we might expect effects to vary by sex and/or conduct problems risk status. Regarding sex, the intervention procedures are likely to appeal particularly to boys given the gendered socialization of competitiveness (Gneezy et al., 2009). In relation to conduct problems risk status, it stands to reason that those whose behavior is already a significant cause for concern would stand to gain the most benefit from the GBG, especially given its emphasis on adaptive socialization processes (e.g., alerting children to, and rewarding them for, meeting social task demands in the classroom; Kellam et al., 2011). Finally, on the basis of these two strands of inquiry, we might also reasonably expect to observe amplified intervention effects at their intersection (e.g., boys exhibiting elevated symptoms of conduct problems; Kellam et al., 1994). However, the nature and magnitude of such effects could feasibly vary by outcome domain, supporting the adoption of a multi-variate approach reported herein.

### 1.1 Using growth curve models to analyze the impact of universal preventive interventions on developmental trajectories

Growth curve models (GCMs) have a long tradition in educational research (e.g., Bryk & Raudenbush, 1987; Goldstein, 1987, Goldstein, 1989; Plewis, 1996, Plewis, 2005, Plewis, 2010; Van der Leeden, 1998) and yield great promise for researching the long-term effects of universal, preventive interventions (Greenberg & Abenavoli, 2017). GCMs allow us to determine the overall trajectories of a group of individuals measured repeatedly, while decomposing the total variance into within and between individuals, as well as further aggregated levels, such as schools. GCMs are especially adept in the analysis of developmental scores because they allow us to measure the systematic and variable individual trajectories of a given developmental measure (or a set of them), typically over a relatively long period and/or at more than two occasions. A relatively large number of data points is advantageous as it allows us to give a more nuanced perspective on the individual trajectories. Put simply, two data points over a number of individuals only allow us to fit a series of straight lines, whereas three or more data points potentially allow for curves to be fitted.

In our specific case, we assumed that children's 4-year trajectories of disruptive behavior, concentration problems, and prosocial behavior are systematic since they follow a pattern over the time period (specified as a reasonably complex function of time), and they are variable because children have varying rates of change across time (growth). These trajectories are also group-specific and outcome-specific since they estimate differences between groups defined by individual characteristics (e.g., sex) or school characteristics (e.g., trial arm), in each of the three developmental outcome measures. Considering all of the above, the research question we sought to address was: What are the effects of the Good Behavior Game on the long-term trajectories of disruptive behavior, concentration problems, and prosocial behavior?

## 2 Method

### 2.1 Design

**Fig. 1:**
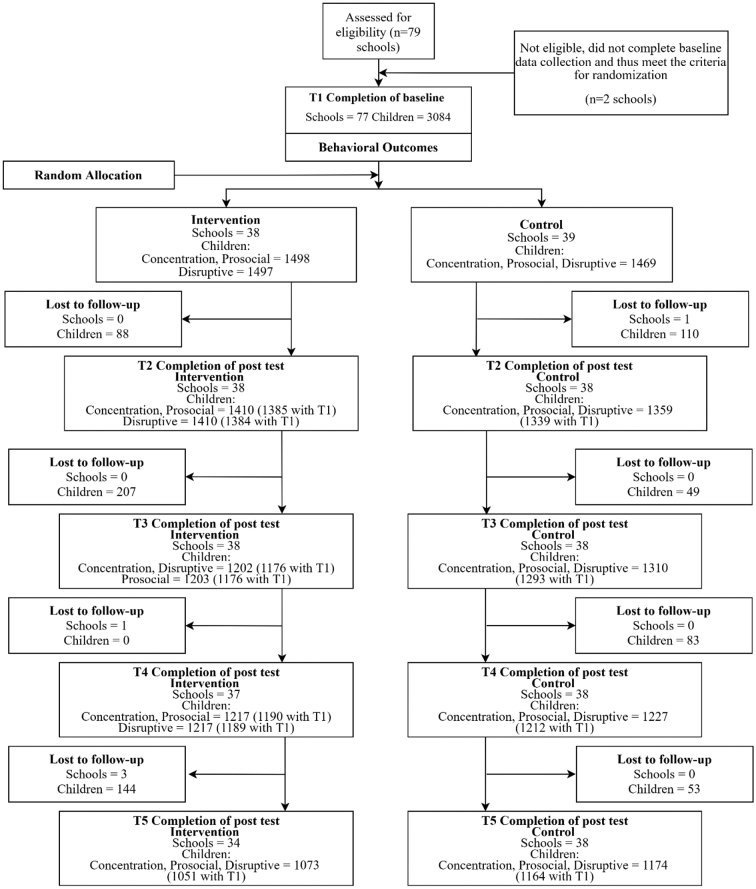
Flow of participants through the study.

A cluster-RCT design was used and is described in more detail elsewhere (Humphrey et al., 2018; trial registration: ISRCTN64152096). Seventy-seven schools were randomly allocated by an independent trial unit to deliver the GBG (intervention) or continue usual practice (control) for a period of two years. A minimization algorithm was used to ensure balance across trial arms with respect to school size and the proportion of children eligible for free school meals. Outcome data were collected at baseline (pre-randomization, Time 1 [T1]) and then annually on four further occasions (Time 2 [T2], Time 3 [T3], Time 4 [T4], Time 5 [T5]). T1 to T3 (Humphrey et al., 2018) represents the period of GBG implementation in the intervention arm of the trial and T3 to T5 represents a clean follow-up phase (Humphrey et al., 2021; i.e., none of the trial sample were exposed to the GBG during this period). Fig. 1 depicts the flow of participants through the study.

Ethical approval was granted by the authors' host institution (The University of Manchester, Ref: 15126). All schools signed a Memorandum of Agreement confirming their willingness to participate. Opt-out consent was sought from parents/caregivers, of whom 68 (2.2%) exercised their right to opt their children out of the trial. Finally, children were provided with information about the study (including their guarantee of anonymity and right to withdraw) and were asked to give their assent to participate; none declined assent or exercised their right to withdraw from the study.

### 2.2 Participants

#### 2.2.1 Schools

Trial schools' composition mirrored that of primary schools in England in respect to size and the proportion of children speaking English as an additional language; however, trial schools contained significantly larger proportions of children with special educational needs and disabilities and children eligible for free school meals, which was used as a proxy to indicate lower income or socio-economic disadvantage (Department for Work and Pensions, 2013), in addition to lower rates of absence and attainment. Intervention and control schools did not differ substantively on any of these characteristics (e.g., intervention schools had an average of 27.56% of children eligible for free school meals, whereas control schools had an average of 24.46%; Humphrey et al., 2018, p. 34).

Within intervention and control schools, 279 classroom teachers of children in the trial cohort participated during the main trial period (T1 to T3), of whom 139 were in control schools and 140 were in GBG treatment schools. Specific to different phases of the trial, 135 classroom teachers taught the trial cohort from T1 to T2 and 144 classroom teachers taught the trial cohort from T2 to T3. Teachers in GBG schools received 2 days of initial training and an additional single day of follow-up training midway through their first year of implementation (approximately 21 h total). This pattern was repeated in the second year of the trial as children moved up into new classes. On-going support for implementation was provided by trained GBG coaches employed by Mentor UK, who in turn were supported by staff at the American Institutes for Research.

#### 2.2.2 Children

At baseline, 3084 children ages 6–7 years from participating schools comprised the trial cohort. No new joiners were allowed to enroll after T1. Those attending intervention and control schools did not differ significantly with respect to sex, free school meals eligibility (FSM), English as an additional language (EAL), or special educational needs and disabilities (SEND; Humphrey et al., 2018). Lost to follow-up numbers are reported in Fig. 1. Lost to follow-up refers to data on outcomes that were not possible to collect. These data are independent of whether a given school has continued or ceased implementation. For example, a school may have discontinued implementation but continued to provide outcome data. Furthermore, some schools may have not returned all surveys in all waves and some may have dropped out of one wave and returned in a subsequent one.

The at-risk subsample was comprised of 485 (15.7%) children scoring in the borderline or abnormal range (i.e., a score of 3 or more out of 10) on the teacher informant-report version of the Strength and Difficulties Questionnaire (SDQ; Goodman, 1997) at baseline. Two hundred seventy-two students attended intervention schools and 213 students attended control schools; 143 students were female and 342 students were male.

### 2.3 Measures

#### 2.3.1 Disruptive behavior, concentration problems, and prosocial behavior

The Teacher Observation of Classroom Adaptation–Checklist (TOCA-C; Koth, Bradshaw, & Leaf, 2009) is a 21-item instrument that provides indices of children's disruptive behavior (nine items), concentration problems (seven items), and prosocial behavior (five items). Teachers read statements about a child (e.g., “pays attention”) and endorse items on a 6-point scale (from *never* to *always*). Evidence has suggested that data derived from the TOCA-C are internally consistent (i.e., all subscales α > 0.86) and have a factor structure that is invariant across gender, race, and age (Koth et al., 2009). Furthermore, Bradshaw, Waasdorp, and Leaf (2012) demonstrated that the data derived from the TOCA-C are sensitive to change in universal preventive interventions. Kourkounasiou and Skordilis (2014) provided further evidence of divergent validity, concurrent validity, and test-retest reliability. We also provide evidence of strict longitudinal measurement invariance in the current study.

#### 2.3.2 Co-variates

Outcome data were supplemented by socio-economic and demographic information obtained from the National Pupil Database and a conduct problems risk status indicator derived from the teacher informant-report version of the SDQ (Goodman, 1997) that was administered at baseline (see *Children* above). School level data were drawn from the Department for Education website. Table 1 provides a summary description of the variables used in the current study.

**Table 1:** Variables used in the current study.

Variable	Description
Disruptive behavior (standardized)	TOCA-C subscale score for disruptive behavior. Time varying continuous outcome. Original scores range 1–6, with higher scores indicating greater disruptive behavior.
Concentration problems (standardized)	TOCA-C subscale score for concentration problems. Time varying continuous outcome. Original scores range 1–6, with higher scores indicating greater concentration problems.
Prosocial behavior (standardized)	TOCA-C subscale score for prosocial behavior. Time varying continuous outcome. Original scores range 1–6, with higher scores indicating greater pro-social behavior.
Trial arm	Nominal time-invariant school-level covariate. Coded 0 = control; 1 = GBG
FSM	Nominal time-invariant pupil-level covariate. Free-school meal eligibility at T1. Coded 0 = Non-FSM; 1 = FSM
Conduct problems risk status	Nominal time-invariant pupil-level variable. “At risk” is defined as scoring 3 or more in the SDQ conduct problems subscale (slightly raised) at T1. Coded 0 = not at risk; 1 = at risk
Sex	Nominal time-invariant pupil-level covariate. Sex as registered at birth and recorded in the National Pupil Database. Coded 0 = female; 1 = male
School FSM	Continuous (standardized) time-invariant school-level covariate. Percentage of pupils eligible for free-school meals at T1.
School size	Continuous (standardized) time-invariant school-level covariate. Number of pupils on roll at T1.

*Note.* FSM = eligible for free school meals.

#### 2.3.3 GBG implementation and the counterfactual

Data derived from an online GBG scoreboard developed by the research team for use by participating teachers indicated that children in the intervention arm were exposed to the GBG for an average of 1066 min between T1 and T3 (*SD* = 719.5). In terms of frequency and duration, teachers played the game approximately twice a week between T1 and T2 and between once and twice a week between T2 and T3; the average game session length in both years was approximately 15 min. Nine GBG schools formally ceased implementation prior to T3, although their dosage data are included in the above estimates. Likewise, their data on outcomes, when provided, were also included in our analyses, hence they were not always “lost to follow-up”.

Other dimensions of implementation were assessed via a structured observation schedule administered annually by a member of the research team. In relation to fidelity, a list of required steps outlined in the GBG manual (Ford et al., 2014) were scored on a binary yes/no scale. Fidelity was high (70%) in both years, indicating that teachers followed most of the prescribed procedures associated with the GBG. Quality was rated on a 5-item scale (each scored 0–2) and was also high (70%) in both years, indicating enthusiastic and engaging delivery. Almost all children in a given class were present when the game was played throughout the main trial period (>95% reach from attendance count). Participant responsiveness was rated on an 8-item scale (each scored 0–2) and indicated that children responded favorably (e.g., correcting their behavior following an infraction) when the game was being played (75% from T1 to T2; 69% from T2 to T3; Humphrey et al., 2018).

In terms of the counterfactual, surveys of usual practice in behavior management (derived from Reupert & Woodcock, 2010) among teachers in control schools revealed that 95% reported that they established and maintained a set of classroom rules, 90% reported communicating clear expectations about rules and children's responsibilities (e.g. through posters), 100% reported that they observed and monitored children's behavior in the classroom, 60% used prizes as rewards for good behavior daily or weekly, and 67% used group rewards daily or weekly (Humphrey et al., 2018). These data appear to indicate relatively low program differentiation; however, as a counterpoint we note that the idea of an untreated control group in the context of school-based preventive interventions has long been regarded as a fantasy (Durlak, 2015).

### 2.4 Analytical strategy

#### 2.4.1 Multivariate multilevel growth curve modeling

Growth curve models are a type of statistical model for repeated measures in which occasions (or time points) are nested within individuals. Under a multilevel modeling framework, the total variance of the outcome of interest is split into variance between occasions (within individuals) and variance between individuals. Time is modeled explicitly in the model as a lower-level (occasions) covariate and its slope is allowed to vary randomly across the higher-level units (individuals); in other words, a growth curve model is a random slopes model. Time can also be treated flexibly to allow for non-linear effects. The multilevel specification of a growth curve model also has the advantage of allowing for further higher levels and multivariate outcomes. The approach presented here is largely equivalent to a latent growth curve modeling approach, with the notable exception that the multilevel specification fits a single (invariant) within-individual variance, which is a reasonable assumption when time is treated flexibly (Goldstein, 2011). This model also assumes uncorrelated within-individual residuals, which is reasonable when the repeated observations are not very close together in time (Goldstein, 2011; Hox et al., 2017).

The model we fit in this paper was a multivariate non-linear growth curve model, the algebraic form of which is provided in Appendix A. The intercept was allowed to vary randomly across children and schools, which allowed us to examine the split of the total variance across time, children, and schools. This model treats time as a cubic polynomial term and allows the slope of the linear term for time (i.e., growth rate) to vary randomly across children. The model was estimated by using the Markov Chain Monte Carlo (MCMC) algorithm as implemented in MLwiN (Rasbash et al., 2020), which we called from within R (R Core Team, 2019) using the package “R2MLwiN” (Zhang et al., 2016). The MCMC chains use the coefficient estimates from the Iterative Generalized Least Squares (IGLS) algorithm as starting values. Overall model fit was evaluated by using the Deviance Information Criterion (DIC; Spiegelhalter et al., 2002). Details of the code can be found in Appendix B.

The multivariate multilevel specification allowed us to measure the effect of the GBG simultaneously on our three outcomes while controlling for the correlation between them (which helps to avoid confounding) and a set of socio-economic, demographic, behavioral, and school-level baseline characteristics. As noted by Troncoso (2019), this specification also allowed us to test cross-outcome hypotheses (e.g., whether the effects of a particular covariate differs across outcomes). A further advantage is that the variance-covariance matrix is efficiently estimated even in the presence of missing data (Goldstein, 2011), rendering it equivalent to full information maximum likelihood (FIML) because it uses all the available information and results are therefore unbiased under the assumption of data Missing at Random (MAR). Our full model preserved 2938 children (95.27%) in 77 schools (100%); time-specific missing values for the outcomes by trial arm are reported in Fig. 1. We fitted a multilevel model for missingness in the full model, which failed to find enough evidence that our main covariates predicted missingness. Details of this procedure are provided in Appendix C.

Even though this is a trial, effect sizes cannot be reported as there is no exact equivalent in the Bayesian framework. Instead, we report standardized coefficients, as well as the Bayes Factor (BF) and Posterior Model Probabilities (PMP) for the GBG effect estimate over time, following Moerbeek's (2019) “informative hypothesis evaluation” approach.

#### 2.4.2 Longitudinal measurement invariance

Item-level TOCA-C analyses were performed to determine the longitudinal invariance of the three subscales, following Grimm et al. (2017) approach. We used the R package “lavaan” (Rosseel, 2012) to run confirmatory factor analyses. Details of the procedure, code, and outputs are available on request. Concisely, the procedure involved replicating the factor structure over the five time points for each subscale of the TOCA-C and fitting increasingly constrained models to determine whether the fit of the measurement model significantly worsened. The first fitted model, termed “configural”, was an unconstrained model with factor loadings, observed variables means, variances, and covariances estimated freely. The configural model (Measurement Model 1) was used as the baseline comparison for three other models. The *weak invariance* model (Measurement Model 2) constrained the factor loadings across time points to equality and the *strong invariance* model (Measurement Model 3) constrained the means of the observed variables to equality across time while keeping the constraints of Measurement Model 2. Finally, the *strict invariance* model (Measurement Model 4) placed additional constraints by setting residual variances to be equal across time. A comparison of the model fit of these models for all outcomes can be seen in Table 2.

**Table 2:** Goodness of fit comparison across longitudinal measurement models for TOCA subscales.

Outcome	Model	CFI	TLI	RMSEA	AIC	BIC	Chi-squared	df	SRMR
Concentration problems	Configural	0.974	0.967	0.045	194,240.1	195,352.3	3376.9	480	0.034
	Weak	0.973	0.968	0.044	194,281.9	195,249.9	3466.8	504	0.037
	Strong	0.970	0.967	0.045	194,559.0	195,358.6	3799.8	532	0.039
	Strict	0.967	0.965	0.047	194,935.1	195,566.4	4232.0	560	0.039
Disruptive behavior	Configural	0.939	0.929	0.048	237,696.2	239,109.0	6619.0	845	0.031
	Weak	0.935	0.927	0.048	238,042.1	239,262.5	7028.9	877	0.044
	Strong	0.928	0.922	0.050	238,709.4	239,713.4	7768.2	913	0.046
	Strict	0.922	0.919	0.051	239,191.0	239,978.6	8321.8	949	0.047
Prosocial behavior	Configural	0.947	0.926	0.059	157,868.7	158,680.3	2435.6	215	0.045
	Weak	0.945	0.929	0.057	157,917.9	158,633.3	2516.8	231	0.048
	Strong	0.940	0.929	0.057	158,112.2	158,707.4	2751.1	251	0.050
	Strict	0.937	0.930	0.057	158,238.6	158,713.6	2917.5	271	0.052

*Note.* CFI = Comparative Fit Index; TLI = Tucker Lewis Index; RMSEA = Root Mean Square Error of Approximation; AIC = Akaike Information Criterion; BIC = Bayesian Information Criterion; df = degrees of freedom; SRMR = Standardized Root Mean Square Residual.

Given the oversensitivity of the Chi-squared test for large samples, we judged the goodness of fit by using the Comparative Fit Index (CFI), Tucker Lewis Index (TLI), Root Mean Square Error Approximation (RMSEA), and Standardized Root Mean Square Residual (SRMR) measures. The freely estimated measurement models (i.e., configural) displayed better goodness of fit measures than the constrained models (i.e., weak, strong, and strict) for all outcomes. Nevertheless, strict invariance models for all outcomes demonstrated acceptable goodness of fit, with CFI and TLI values close to 0.95 (Hu & Bentler, 1999). The largest departure from this was the disruptive behavior subscale, which yielded the lowest CFI and TLI values (0.922 and 0.919, respectively); however, RMSEA and SRMR values were still below conventional thresholds (i.e., < 0.06 and < 0.08, respectively; Hu & Bentler, 1999), as was also the case for concentration problems and prosocial behavior. In conclusion, the assumption of strict invariance was justifiable since the models for all three outcomes showed adequate fit. This allowed us to use the TOCA-C composite scores because strict invariance avoids confounding change in scores over time with changes in reliability of the items (Newsom, 2015).

## 3 Results

### 3.1 Observed trajectories over time

Table 3 displays the means and standard deviations over time by trial arm. The means for concentration problems and prosocial behavior tended to decrease each year, with standard deviations remaining relatively stable. The opposite occurred with disruptive behavior as observed means tended to increase with time.

**Table 3:** Summary of descriptive statistics of the observed outcomes over time.

Time	Concentration problems				Disruptive behavior				Prosocial behavior			
	Control		GBG		Control		GBG		Control		GBG	
	Mean	SD	Mean	SD	Mean	SD	Mean	SD	Mean	SD	Mean	SD
1	2.548	1.146	2.602	1.130	1.612	0.812	1.709	0.810	4.946	0.917	4.893	0.875
2	2.657	1.134	2.576	1.126	1.644	0.745	1.761	0.798	4.910	0.920	4.844	0.924
3	2.495	1.129	2.548	1.133	1.647	0.837	1.740	0.856	4.932	0.952	4.808	0.930
4	2.432	1.135	2.437	1.178	1.706	0.789	1.747	0.854	4.917	0.963	4.915	0.960
5	2.392	1.174	2.352	1.148	1.740	0.863	1.732	0.840	4.842	0.981	4.916	0.953

*Note.* SD = standard deviation.

Table 3 provides indications of small differences by trial arm. Children in GBG schools tended to have lower mean concentration problems (2.352) and disruptive behavior (1.732) than those in control schools (2.392 and 1.74, respectively) by the end of the period of study, having started off at marginally higher means (2.602 and 1.709 in GBG, as opposed to 2.548 and 1.612 in control). The opposite trend occurred for prosocial behavior where children in GBG schools began with slightly lower means (4.893) than the control group (4.946) but tended to surpass them by T5 (4.916 in GBG and 4.842 in control). However, these time trends are only descriptive and they most likely obscure underlying patterns of variation that we unveil in the following sections.

### 3.2 Variation across outcomes and levels

We fitted an unconditional means model (i.e., empty multivariate multilevel model) to assess the weight of the contribution of each level to the total variation in concentration problems, disruptive behavior, and prosocial behavior. This is a model in which no explanatory variables are added as it is only used to decompose the total variance into variance within children (Level 1), between children (Level 2), and between schools (Level 3). Although no explanatory variables are included, the model does control for the correlation between outcomes; hence, the means and variances presented in Table 4 are more accurate than the observed mean trajectories presented in Table 3.

**Table 4:** Unconditional means multivariate multilevel model for concentration problems, disruptive behavior, and prosocial behavior (standardized).

Fixed part	Post. mean	SD
Intercept concentration	0.026	0.028
Intercept disruptive	0.038	0.032
Intercept prosocial	−0.017	0.033

Level	Random part	Post. mean	SD	Correlation
Between schools	Variance (intercept concentration)	0.040	0.010	–
	Covariance (concentration, disruptive)	0.039	0.010	0.817
	Variance (intercept disruptive)	0.056	0.013	–
	Covariance (concentration, prosocial)	−0.045	0.011	−0.860
	Covariance (disruptive, prosocial)	−0.047	0.012	−0.755
	Variance (Intercept prosocial)	0.069	0.014	–
Between children	Variance (Intercept concentration)	0.572	0.018	–
	Covariance (concentration, disruptive)	0.430	0.015	0.750
	Variance (Intercept disruptive)	0.574	0.018	–
	Covariance (concentration, prosocial)	−0.389	0.014	−0.826
	Covariance (disruptive, prosocial)	−0.397	0.014	−0.842
	Variance (Intercept prosocial)	0.387	0.014	–
Within children	Variance (Intercept concentration)	0.406	0.006	–
	Covariance (concentration, disruptive)	0.148	0.004	0.367
	Variance (Intercept disruptive)	0.400	0.006	–
	Covariance (concentration, prosocial)	−0.264	0.005	−0.553
	Covariance (disruptive, prosocial)	−0.239	0.005	−0.505
	Variance (Intercept prosocial)	0.560	0.008	–

*Note.* Deviance information criterion = 97,603.35.

The correlations between outcomes display notable differences across levels. Concentration problems and disruptive behavior were strongly and positively associated at the level of children and schools; however, they were more weakly associated at the within-child level. This may indicate that time-invariant factors (i.e., invariant child characteristics) played a more important role in the relationship between these outcomes, especially concentration problems and disruptive behavior. This is further supported by the variance composition presented in Table 5.

**Table 5:** Variance partitioning of the unconditional means model for concentration problems, disruptive behavior, and prosocial behavior.

Outcome	Within children	Between children	Between schools
Concentration problems	39.88%	56.23%	3.88%
Disruptive behavior	38.81%	55.72%	5.47%
Prosocial behavior	55.12%	38.05%	6.82%

It is worth noting that the variation in prosocial behavior was greater within children than between children (55.12% and 38.05%, respectively), as opposed to the variation in concentration problems (39.88% and 56.23%, respectively) and disruptive behavior (38.81% and 55.72%, respectively). Even though differences between children comprised a large proportion of the total variation in prosocial behavior, it appears as if this developmental process was more markedly an individual process over time. Judging by the proportion of variance that was attributable to differences between schools, prosocial behavior (6.82%) appeared to be slightly more driven by the wider school context than concentration problems (3.88%) and disruptive behavior (5.47%).

### 3.3 A conditional growth model for the effect of the GBG on children's trajectories of disruptive behavior, concentration problems, and prosocial behavior

**Fig. 2:**
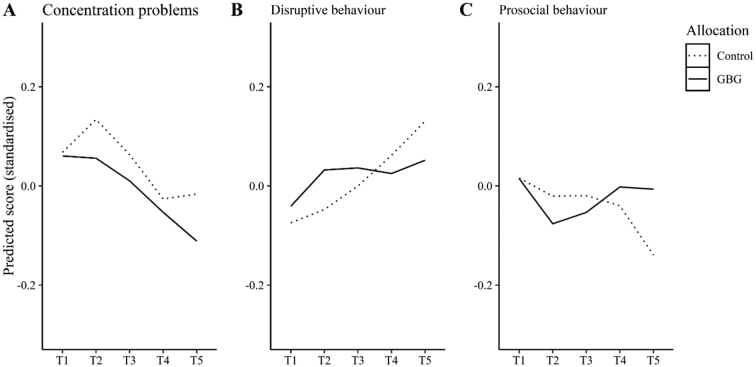
Predicted standardized scores for concentration problems, disruptive behavior and prosocial behavior by trial arm.

Fig. 2 shows quite distinct average temporal trajectories for all three outcomes by trial arm. Fig. 2A indicates that concentration problems tended to decrease over time, but they did so more rapidly for children in GBG schools. Fig. 2B highlights that disruptive behavior increased over time with those in the intervention group apparently making shallower increases and a lower expected average by the end of the study period. Fig. 2C reveals a downward trajectory in prosocial behavior; however, those in GBG schools remained roughly at the same average they started at and were apparently higher than their counterparts in usual practice schools.

To assess these trends more robustly, we began by fitting an intermediate unconditional model and found that time was a relevant factor in all three processes before controlling for covariates, although some differences were noted. Concentration problems and prosocial behavior exhibited strong evidence of non-linear (cubic) trends; however, the former increased over time and the latter decreased over time. Disruptive behavior also displayed an increase over time; however, there was not enough evidence to assert it was a non-linear trend. We kept these non-linear terms for all outcomes in the subsequent models as they do display better overall fit (assessed via deviance information criterion comparison) than other intermediate models without them. Full details of these intermediate models are available on request. In Table 6, the fixed part of the full, final model is displayed (the random part of this model is available in Appendix D). Children in GBG schools demonstrated a notable decrease in concentration problems over time (posterior mean = −0.151; *SD* = 0.061; 95% credible intervals = −0.270, −0.033); each year, those in the intervention arm of the trial displayed a mean linear decrease of 0.15 *SD* with respect to the previous year, as compared to their counterparts in control schools, although this effect changed across the study period. This trend is more easily interpreted by inspecting Fig. 2A.

**Table 6:** Fixed-effects parameters of the full multivariate multilevel non-linear growth curve model for concentration problems, prosocial behavior, and disruptive behavior (standardized).

Parameter^a^	Concentration problems				Disruptive behavior				Prosocial behavior			
	Post. mean	SD	95% CI		Post. mean	SD	95% CI		Post. mean	SD	95% CI	
Intercept	−0.368	0.043	−0.451	−0.284	−0.496	0.043	−0.579	−0.412	0.353	0.050	0.256	0.452
time	0.174	0.043	0.090	0.258	0.016	0.042	−0.066	0.098	−0.075	0.051	−0.174	0.024
time squared	−0.128	0.028	−0.182	−0.073	0.013	0.027	−0.040	0.066	0.048	0.033	−0.016	0.112
time cubed	0.020	0.005	0.011	0.029	−0.001	0.004	−0.010	0.008	−0.010	0.005	−0.020	0.001
GBG	0.011	0.061	−0.108	0.131	0.072	0.061	−0.049	0.192	−0.020	0.072	−0.163	0.120
time*GBG	−0.151	0.061	−0.270	−0.033	0.109	0.059	−0.007	0.224	−0.102	0.071	−0.242	0.038
time squared*GBG	0.096	0.039	0.019	0.173	−0.073	0.038	−0.148	0.001	0.051	0.046	−0.039	0.142
time cubed*GBG	−0.016	0.007	−0.029	−0.003	0.010	0.006	−0.003	0.022	−0.004	0.008	−0.019	0.011
At risk	1.108	0.104	0.904	1.310	1.517	0.086	1.349	1.686	−1.336	0.091	−1.515	−1.157
FSM	0.262	0.046	0.173	0.351	0.190	0.037	0.117	0.263	−0.216	0.040	−0.294	−0.139
School size	−0.041	0.033	−0.105	0.024	−0.062	0.037	−0.134	0.011	−0.001	0.043	−0.086	0.083
School FSM	0.029	0.033	−0.036	0.092	−0.002	0.035	−0.071	0.067	−0.003	0.041	−0.083	0.078
GBG and FSM	−0.022	0.062	−0.143	0.099	−0.059	0.050	−0.159	0.039	−0.004	0.054	−0.109	0.102
GBG and at risk	−0.164	0.130	−0.419	0.091	−0.205	0.108	−0.416	0.007	0.377	0.115	0.152	0.602
Male	0.390	0.039	0.313	0.467	0.232	0.032	0.169	0.295	−0.184	0.034	−0.251	−0.117
GBG and male	0.002	0.056	−0.107	0.112	0.013	0.046	−0.077	0.103	−0.036	0.049	−0.132	0.059
Male and at risk	−0.085	0.119	−0.319	0.150	0.176	0.098	−0.017	0.369	0.247	0.104	0.042	0.453
GBG, male and at risk	0.114	0.155	−0.190	0.417	0.072	0.128	−0.179	0.322	−0.203	0.136	−0.469	0.063
GBG*school size	0.096	0.054	−0.009	0.203	0.032	0.061	−0.088	0.153	−0.067	0.071	−0.208	0.071
GBG*school FSM	−0.004	0.046	−0.094	0.086	0.046	0.050	−0.052	0.143	0.016	0.058	−0.098	0.129

*Note.* GBG = Good Behavior Game; FSM = eligible for free school meals.

a Parameters were obtained via Markov Chain Monte Carlo (MCMC) estimation with Gibbs sampling using 3 parallel chains of length 100,000 and a burn-in period of 1000 (storing all iterations). All fixed-effects parameters have an effective sample size (ESS) of at least 4000. Deviance information criterion = 73,211.344. The model uses diffuse prior distributions as described in Browne (2019). Trajectories mix well with approximately normally-distributed posteriors; however, they are not presented here as they exceed the scope of this paper. Full details are available on request.

In Table 7, we present the Bayes Factor (BF) estimates and their corresponding posterior model probabilities (PMP). For all outcomes, the informative hypotheses are split into two options: (a) outcomes for GBG participants were better than the outcomes of the control group (H1) and (b) outcomes for GBG participants were equal to or worse than the outcomes of the control group (H2). In the case of concentration problems and disruptive behavior, if the GBG produces better outcomes (H1), then we would expect the difference between the mean of the control group and the trial arm to be positive (H1: Control – GBG > 0), which would indicate that GBG participants had less concentration problems and disruptive behavior than the control group. Conversely, for prosocial behavior, our informative hypothesis was that if GBG produces better outcomes, then the difference between the mean of the control group and the trial arm was expected to be negative (H1: Control – GBG < 0). The Bayes Factor estimates for informative hypothesis H1 is BF_1_ and consequently the Bayes Factor estimates for informative hypothesis H2 (the complement of H1) is BF_2_. Their corresponding posterior model probabilities (PMP_1_ and PMP_2_) indicate the estimated probability of the hypothesis being true, given the observed data. An additional indication of the strength of the evidence is given by the Bayes Factor estimate of H1 over H2 (BF_1,2_), which provides a measure of how likely H1 is in comparison with H2. If BF_1,2_ is over 1, then H1 is more likely than H2; if it is less than 1, then H1 is less likely than H2 and if it is 1, both hypotheses are equally likely. Following van Doorn et al. (2020), BF_1,2_ estimates between 1 and 3 would be considered “weak” evidence in favor of H1 over H2; values between 3 and 10 would be considered “moderate” evidence; and “strong” evidence would be backed up by estimates over 10. Nevertheless, Moerbeek (2019) cautioned against the stringent use of interpretative rules for Bayes Factor estimates and so these are used only as broad guides here.

**Table 7:** Bayes factors and posterior model probabilities for the difference in outcomes between children in the trial and the control group over time.

Outcome	Time	Control	GBG	Difference	BF_1_	BF_2_	BF_1,2_	PMP_1_	PMP_2_
Concentration problems	1	−0.367	−0.357	−0.011	0.863	1.137	0.759	0.432	0.568
	2	−0.301	−0.362	0.061	1.681	0.319	5.264	0.84	0.16
	3	−0.372	−0.407	0.035	1.441	0.559	2.58	0.721	0.279
	4	−0.461	−0.470	0.009	1.117	0.883	1.264	0.558	0.442
	5	−0.451	−0.528	0.077	1.772	0.228	7.789	0.886	0.114
Disruptive behavior	1	−0.495	−0.425	−0.07	0.255	1.745	0.146	0.127	0.873
	2	−0.468	−0.352	−0.115	0.064	1.936	0.033	0.032	0.968
	3	−0.421	−0.348	−0.072	0.247	1.753	0.141	0.123	0.877
	4	−0.359	−0.360	0.001	1.004	0.996	1.007	0.502	0.498
	5	−0.289	−0.333	0.044	1.469	0.531	2.77	0.735	0.265
Prosocial behavior	1	0.353	0.334	0.019	0.791	1.209	0.655	0.396	0.604
	2	0.317	0.243	0.074	0.300	1.700	0.177	0.150	0.850
	3	0.318	0.266	0.053	0.459	1.541	0.298	0.230	0.770
	4	0.298	0.318	−0.019	1.204	0.796	1.512	0.602	0.398
	5	0.198	0.312	−0.114	1.862	0.138	13.524	0.931	0.069

Notes. Informative Hypothesis 1: Control > GBG (for concentration problems and disruptive behavior); Control < GBG (for prosocial behavior). Informative Hypothesis 2: Control ≤ GBG (for concentration problems and disruptive behavior); Control ≥ GBG (for disruptive behavior). BF_1_ = Bayes Factor for Informative Hypothesis 1. BF_2_ = Bayes Factor for Informative Hypothesis 2. BF_1,2_ = Bayes Factor for Informative Hypothesis 1 over Informative Hypothesis 2. PMP_1_ = Posterior Model Probability for Informative Hypothesis 1. PMP_2_ = Posterior Model Probability for Informative Hypothesis 2.

Table 7 shows higher posterior model probabilities supporting the informative hypothesis that GBG children had less concentration problems (H1) from T2 onwards, with moderate evidence (BF_1,2_ > 5) of better GBG outcomes at T2 and T5. We did not find reliable evidence of an intervention effect on trajectories of disruptive behavior or prosocial behavior, with 95% credible intervals of the posterior mean crossing zero for both outcomes (Table 6) and only weak evidence in favor of H1 over H2 arising from the BF estimates (BF_1,2_ < 3) in Table 7. Nevertheless, Table 7 also shows strong evidence (BF_1,2_ = 13.52) supporting the hypotheses of better outcomes for GBG pupils in prosocial behavior at T5, with a notably higher posterior model probability (PMP_1_ = 0.931) than the alternative (PMP_2_ = 0.069).

Subgroup effects were modeled as main effects rather than in interaction with time in the interests of parsimony (e.g., doing so would require seven additional interaction terms to be introduced to an already complex model) and interpretation of other findings (e.g., doing so would affect the meaning of the main time * GBG interaction such that it would refer to females not at risk in the intervention arm, which was not our intention). At-risk children (e.g., those with elevated symptoms of conduct problems at T1) in the intervention arm (*n* = 272) of the trial recorded a noteworthy higher posterior mean for prosocial behavior when compared to their counterparts in the control group (*n* = 213) with a posterior mean estimate of 0.377 (*SD* = 0.115; 95% credible intervals = 0.152, 0.602), which implies that they are expected to have a prosocial score nearly two fifths of a standard deviation higher than those in the control group across all time points. This finding provides robust evidence that the GBG contributed to improved prosocial behavior outcomes among at-risk children. However, we did not find equivalent effects for disruptive behavior or concentration problems, with credible intervals of the posterior mean crossing zero for both outcomes. Finally, we found no reliable evidence of differential intervention effects for males (control *n* = 837; GBG *n* = 786) or indeed at-risk males (control *n* = 162; GBG *n* = 180) for any of our three outcomes.

## 4 Discussion

In our main report on the first trial of the GBG in England (Humphrey et al., 2018), we examined its effects on reading and behavioral outcomes in the short-term using point-in-time estimates, finding no evidence of its efficacy. The current study built upon and extended this work by using growth curve models as opposed to point-in-time estimates to examine long-term, as opposed to short-term, main and subgroup (sex and conduct problems risk status, with effects examined separately *and* in combination, as opposed to solely the latter) effects of the intervention on children's behavioral outcomes. We fitted a multivariate multilevel growth curve model that examined trajectories that spanned two years of implementation and two additional years of follow-up. Our analyses revealed that the intervention altered trajectories of concentration problems, with those exposed to the GBG experiencing a mean linear decrease of 0.151 *SD* with respect to the previous year (and strong support for better outcomes at T5), relative to their counterparts in control schools (although this effect changes through time, as evidenced by the polynomial terms for time). However, we did not find reliable evidence of an intervention effect on trajectories of disruptive behavior or prosocial behavior, with the notable exception of higher posterior probabilities supporting better outcomes for GBG at T5. In terms of subgroup moderator effects, we found robust evidence that the intervention benefited those children with elevated symptoms of conduct problems at baseline, with a 0.377 *SD* difference between children in GBG and control schools across time. However, we did not find equivalent effects for disruptive behavior or concentration problems. Finally, we found no reliable evidence of differential intervention effects for males, or indeed at-risk males, for any of our three outcomes.

Our model provided robust evidence that the GBG influences the trajectory of children's concentration problems over time. The direction of this effect is consistent with both the theorized effects of the intervention (Chan et al., 2012) and developmental trends in children's capacity to pay attention, stay on task, and resist distractions during the elementary school years (e.g., Best et al., 2009). Thus, although we observed a general trend indicative of reductions in concentration problems over four years, a notably sharper decrease was evident among children in the intervention arm of the trial who were exposed to the GBG for the first two years of this period (see Fig. 2A). These results align with those of Leflot et al. (2010) who identified a positive effect of the GBG on children's on-task behavior and also align with van Lier et al. (2004) who demonstrated that the intervention impacted developmental trajectories of attentional difficulties. Our findings extend the work of these authors in important ways. In the case of the former, we show the impact on developmental trajectories as opposed to point-in-time estimates. In the case of the latter, although these authors *did* focus on developmental trajectories, their data points only covered the period of GBG implementation, as opposed to the longer-term follow-up period included here.

The other notable intervention effect identified in our model related to improvements in prosocial behavior among at-risk children. This finding mirrors that of Jiang et al. (2018) who identified a subgroup moderator effect on high-risk students' prosocial behavior in their GBG trial, albeit with similar distinctions to those noted above (e.g., point-in-time estimation and time period), alongside different criteria for at-risk status (which was defined using the borderline/abnormal range scores for prosocial behavior as opposed to conduct problems). This effect is particularly noteworthy given the general downward trend in prosocial behavior observed in the sample over the course of the study (see Fig. 2C). It is also interesting to consider in the context of the other (null) outcomes for this subgroup; it might suggest, for example, that prosocial behavior is more malleable and amenable to a relatively low intensity intervention than disruptive behavior.

Our analyses revealed no unequivocal trial effects in relation to disruptive behavior. One possible explanation is that teachers implementing the GBG were much more sensitized to the types of behaviors (e.g., talking out, getting out of seat, touching others, being disobedient or aggressive) being assessed in this particular subscale of the TOCA-C given their central focus in the initial training and subsequent delivery (e.g., infractions associated with the four classroom rules related to acts of disruption; by contrast, there is much less direct focus on concentration and prosocial behavior). In tentative support of this proposition, Fig. 2B illustrates that at T1, which is when all teachers who completed ratings were naïve to the GBG, differences between intervention and control arms were negligible. However, ratings at T2 and T3, which is when teachers in the intervention arm were trained and implementing the GBG, their ratings indicated *higher* rates of disruptive behavior. In the follow-up period (i.e., T4 and T5), teachers providing ratings of children's behaviors were again naïve to the GBG, and it is in this period that we see the disruptive behavior trajectories of the two trial groups cross over, with relative stability in the GBG arm contrasted with increased disruptive behavior among children in the control arm. Future research could address this issue using independent observational data rather than teacher ratings.

### 4.1 Strengths and limitations

The current study benefits from several strengths that contribute to the security of the findings reported herein. We used a cluster-randomized design and adopted an analytical technique that took account of the hierarchical and clustered nature of the dataset, thus allowing us to model the long-term developmental trajectories of our three outcome variables. This multivariate multilevel non-linear growth curve model is particularly well-suited to researching the effects of universal preventive interventions, whose effects may take time to emerge (Greenberg & Abenavoli, 2017). The trial was large and well powered. Attrition was within acceptable limits and missing data were handled using all available information (see Method section). Balance on observables in the analysis was good, with negligible differences between trial arms for children's outcomes at baseline. The use of cluster randomization minimized the possibility of contamination and the allocation procedure itself was conducted independently of the evaluation team by a trial unit. The outcome measure used (i.e., TOCA-C) is psychometrically robust (Koth et al., 2009) and has been demonstrated to be sensitive to change in previous trials of universal preventive interventions (e.g., Bradshaw et al., 2012).

However, there are also several limitations that should be acknowledged. First, out of necessity, teachers providing ratings via the TOCA-C were not blinded to trial allocation status. This introduces the possibility the effects identified are the result of biased ratings. However, this seems quite unlikely given the relative preponderance of null findings (e.g., for disruptive behavior). Furthermore, it should be noted that ratings on only two of the five data points (i.e., T2, T3) were provided by teachers directly involved in implementation of the GBG. Second, we did not assess compliance effects. In the context of this GBG trial, we understand compliance as surpassing a given threshold of dosage due to the low variability in fidelity (e.g., the median or the 75th percentile). As noted earlier, nine of the 38 schools in the trial arm ceased implementation prior to the end of the main trial period (T3). Thus, although our study conforms to the ‘analyze as you randomize’ principle that is designed to provide an unbiased estimate (Gupta, 2011), we cannot rule out the possibility that the presence and magnitude of intervention effects reported here is attenuated by lack of intervention compliance (e.g., dosage variability) among some schools. In other words, we may have observed more and larger intervention effects had the game been played with greater frequency and/or had several schools not discontinued implementation prior to T3. Nevertheless, given that dosage is only observed in the intervention arm, the statistical approach to address this in combination with longitudinal outcome trajectories requires substantial work exceeding the scope of this study. Finally, although the overall trial sample size was very large, the same cannot not be said for our subsample of at-risk boys (11% of overall sample; *n* = 342). Thus, it may well be that the impact of the GBG was too small to be ascertained in this subgroup for this specific sample due to loss of statistical power. In other words, assuming constant sample size, a larger difference or smaller within-group variation would have had to be recorded.

### 4.2 Implications

This study provides robust evidence that the GBG can be used as an efficacious means through which to alter developmental trajectories of children's concentration problems. The ability to pay attention, stay on task, and resist distractions are key social task demands in the classroom context that yield powerful concurrent and prospective benefits in learning and attainment throughout childhood and adolescence (Breslau et al., 2010). Hence, even the relatively small gains (as judged by conventional effect size standards, although we note the growing resistance to reflexive reference to these; e.g., Tanner-Smith et al., 2018) over time evidenced here can be considered practically significant. This is particularly the case given the relatively low input required to bring about these gains; recall that teachers spent an average of only 30 min per week playing the GBG, and this was further reduced in the second year of implementation. It should also be noted that because the game itself is designed to be played during a normal classroom activity, it produces relatively little displacement of academic curriculum time (Ford et al., 2014).

The observed improvements in prosocial behavior among children in GBG schools considered to be at risk by virtue of their emergent conduct problems is also significant from a practical perspective. Life-course research indicates that childhood conduct problems, particularly among boys, are associated with a two-to-threefold increase in early adulthood public sector costs (mainly via the criminal justice system) and significantly higher rates of unemployment (D'Amico et al., 2014; Knapp et al., 2011). Middle childhood is a particularly critical window for intervention in this regard. Notwithstanding the lack of impact on disruptive behavior for this subgroup, which may be more resistant to low intensity intervention and are likely driven by factors beyond the reach of schools when deeply entrenched (Moffitt & Scott, 2008), the moderate observed improvements in behavorial indicators of empathy, compassion, and other facets of prosocial behavior yield great promise given their primacy in terms of children's social adaptational status.

From a methodological standpoint, the current study demonstrates the value and utility of applying growth curve modeling to research the effects of universal preventive interventions. Furthermore, our findings align directly with Greenberg and Abenavoli's (2017) argument that trials of such interventions should always be designed with longer-term follow-up built in from the outset. Given the inevitable variability in implementation, however, an interesting avenue for future research in this space is to develop and make use of fused approaches in which the relationship between intervention compliance and outcome trajectories are modeled. Accordingly, at the time of writing, we are working on ways to implement a statistical approach that combines complier average causal effect estimation and growth curve modeling.

## 5 Conclusion

This study has demonstrated the impact of the Good Behavior Game on children's developmental trajectories of concentration problems, in addition to resulting in notable improvements in prosocial behavior among those with elevated conduct problems. In doing so, it has also highlighted the value and utility of growth curve modeling of intervention effects and including data points that extend well beyond the conclusion of a given period of implementation. Thus, in several ways we have shown that playing the “long game” may come with benefits.

## Funding

This work was supported by the 10.13039/100012343Education Endowment Foundation (no grant number), the 10.13039/501100000272National Institute for Health Research (grant number 14/52/38), and the Economic and Social Research Council (grant number ES/V011243/1). The corresponding author is also affiliated to the Scottish Centre for Administrative Data Research (SCADR), which is part of the Administrative Data Research UK (ADR UK) partnership, funded by the Economic and Social Research Council.

## Declarations of interest

None.

## Figures and Tables

**Figure:**
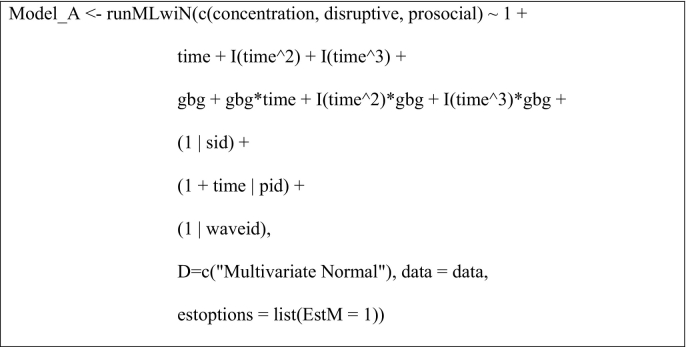


**Figure:**
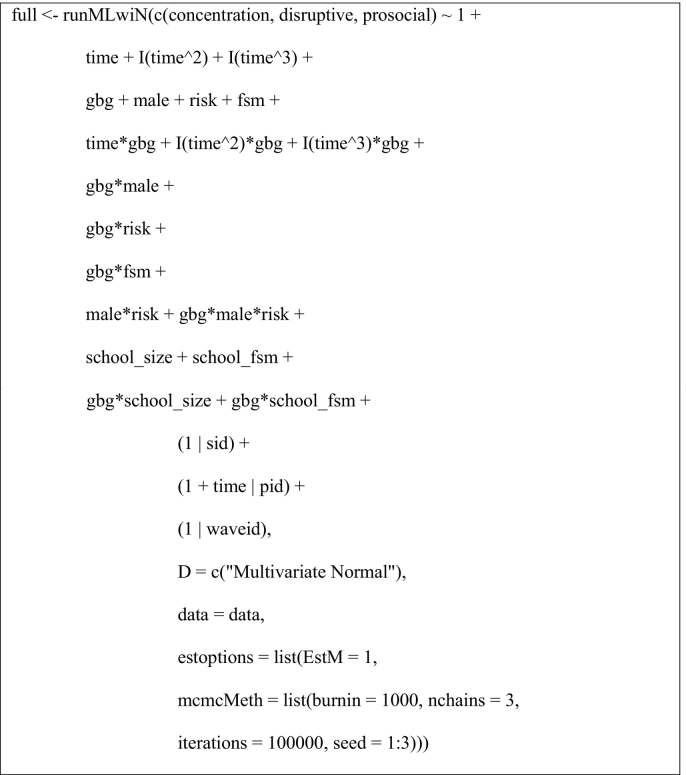


**Table C.1:** Binary logistic multilevel model of children nested within schools for missingness in the full model (Table 6).

Fixed part	Coef.	S.E.	95% Conf. Int.	
Intercept	−4.701	0.418	−5.520	−3.882
Male	0.187	0.363	−0.524	0.899
FSM	0.890	0.687	−0.456	2.235
At risk	−0.179	0.510	−1.178	0.821
Trial (if GBG)	−0.755	0.610	−1.950	0.441
School size	0.145	0.194	−0.236	0.525
School FSM	0.065	0.211	−0.349	0.479

Random part				
Variance (Intercept)	0.276			
VPC	0.077			

Note. Bayesian Information Criterion (BIC) = 3844.33. GBG = Good Behavior Game; FSM = eligible for free school meals; VPC = Variance Partitioning Coefficient. Parameters were obtained via Maximum Likelihood using Mplus version 8 (Muthén & Muthén, 2017) called from the R package “MplusAutomation” (Hallquist & Wiley, 2018).

**Table D.1.** Random part of the full multivariate multilevel non-linear growth curve model for concentration problems, prosocial behavior and disruptive behavior.

Level	Parameter	Post. mean	S.D.	95% Cred. Int.		ESS
Between schools	Variance (Intercept concentration)	0.028	0.007	0.017	0.044	24,749
	Covariance (Concentration, Disruptive)	0.026	0.007	0.015	0.041	31,158
	Variance (Intercept disruptive)	0.040	0.008	0.026	0.059	40,698
	Covariance (Concentration, Prosocial)	−0.034	0.008	−0.052	−0.021	35,511
	Covariance (Disruptive, Prosocial)	−0.033	0.008	−0.052	−0.019	46,303
	Variance (Intercept prosocial)	0.058	0.012	0.039	0.085	54,959
Between children	Variance (Intercept concentration)	0.363	0.017	0.332	0.397	11,807
	Covariance (Concentration, Disruptive)	0.115	0.010	0.097	0.135	5919
	Variance (Intercept disruptive)	0.129	0.009	0.112	0.148	4066
	Covariance (Concentration, Prosocial)	−0.178	0.012	−0.203	−0.155	6002
	Covariance (Disruptive, Prosocial)	−0.100	0.009	−0.118	−0.083	3754
	Variance (Intercept prosocial)	0.148	0.012	0.125	0.172	3180
	Covariance(Intercept concentration, time concentration)	−0.002	0.004	−0.010	0.005	3970
	Covariance(Intercept disruptive, time concentration)	0.016	0.003	0.011	0.021	2668
	Covariance(Intercept prosocial, time concentration)	−0.003	0.003	−0.008	0.004	2455
	Variance(time concentration)	0.008	0.001	0.006	0.011	2216
	Covariance(Intercept concentration, time disruptive)	0.016	0.004	0.009	0.023	5166
	Covariance(Intercept disruptive, time disruptive)	0.027	0.003	0.021	0.031	1928
	Covariance(Intercept prosocial, time disruptive)	−0.014	0.003	−0.019	−0.007	2576
	Covariance(time concentration, time disruptive)	0.006	0.001	0.004	0.008	2834
	Variance(time disruptive)	0.014	0.001	0.012	0.017	3267
	Covariance(Intercept concentration, time prosocial)	−0.014	0.004	−0.021	−0.006	3923
	Covariance(Intercept disruptive, time prosocial)	−0.020	0.003	−0.025	−0.014	2276
	Covariance(Intercept prosocial, time prosocial)	0.017	0.003	0.010	0.022	1596
	Covariance(time concentration, time prosocial)	−0.005	0.001	−0.008	−0.003	2042
	Covariance(time disruptive, time prosocial)	−0.009	0.001	−0.012	−0.007	2943
	Variance(time prosocial)	0.011	0.002	0.008	0.014	2222
Within children	Variance (Intercept concentration)	0.382	0.006	0.370	0.394	12,395
	Covariance (Concentration, Disruptive)	0.136	0.004	0.128	0.145	14,135
	Variance (Intercept disruptive)	0.361	0.006	0.350	0.372	14,503
	Covariance (Concentration, Prosocial)	−0.251	0.006	−0.262	−0.240	13,504
	Covariance (Disruptive, Prosocial)	−0.213	0.005	−0.223	−0.202	14,738
	Variance (Intercept prosocial)	0.531	0.008	0.516	0.547	16,761

## Appendix A Algebraic form of the model

Equation A.1 follows the general multilevel notation as described in Goldstein (2011).

$$\begin{array}{l} y_{ijk}=\beta_{01}z_{1ijk}+\beta_{02}z_{2ijk}+\beta_{03}z_{3ijk}+\beta_{11}z_{1ijk}t_{ijk}+\beta_{12}z_{2ijk}t_{ijk}+\beta_{13}z_{3ijk}t_{ijk}+\beta_{21}z_{1ijk}t_{ijk}^{2}\\ +\beta_{22}z_{2ijk}t_{ijk}^{2}+\beta_{23}z_{3ijk}t_{ijk}^{2}+\beta_{31}z_{1ijk}t_{ijk}^{3}+\beta_{32}z_{1ijk}t_{ijk}^{3}+\beta_{33}z_{3ijk}t_{ijk}^{3}\\ +\beta_{41}z_{1ijk}GBG_{k}+\beta_{42}z_{2ijk}GBG_{k}+\beta_{43}z_{3ijk}GBG_{k}+\beta_{51}z_{1ijk}GBG_{k}t_{ijk}\\ +\beta_{52}z_{2ijk}GBG_{k}t_{ijk}+\beta_{53}z_{3ijk}GBG_{k}t_{ijk}+\beta_{61}z_{1ijk}GBG_{k}t_{ijk}^{2}\\ +\beta_{62}z_{2ijk}GBG_{k}t_{ijk}^{2}+\beta_{63}z_{3ijk}GBG_{k}t_{ijk}^{2}+\beta_{71}z_{1ijk}GBG_{k}t_{ijk}^{3}+\beta_{72}z_{2ijk}GBG_{k}t_{ijk}^{3}\\ +\beta_{73}z_{3ijk}GBG_{k}t_{ijk}^{3} \end{array}$$

$$\begin{array}{l} \beta_{01ijk}=\beta_{01}+v_{01k}+u_{01jk}+e_{01ijk}\\ \beta_{02ijk}=\beta_{02}+v_{02k}+u_{02jk}+e_{02ijk} \end{array}$$

$$\beta_{03ijk}=\beta_{03}+v_{03k}+u_{03jk}+e_{03ijk}$$

$$\begin{array}{l} \beta_{11j}=\beta_{11}+u_{11j}\\ \beta_{12j}=\beta_{12}+u_{12j}\\ \beta_{13j}=\beta_{13}+u_{13j} \end{array}$$

*where*

$$z_{1ijk}=\left\{\begin{array}{cc} 1 & \text{if concentration}\\ 0 & \text{otherwise} \end{array}\right\},\;z_{2ijk}=\left\{\begin{array}{cc} 1 & \text{if prosocial}\\ 0 & \text{otherwise} \end{array}\right\},\;\;z_{3ijk}=\left\{\begin{array}{cc} 1 & \text{if disruptive}\\ 0 & \text{otherwise} \end{array}\right\}$$

$$\left[\begin{array}{c} v_{01k}\\ v_{02k}\\ v_{03k} \end{array}\right]∼MVN(0,\Omega_{v})=\left[\begin{array}{ccc} \sigma_{v01}^{2} & \sigma_{v01,02} & \sigma_{v01,03}\\ \sigma_{v01,02} & \sigma_{v02}^{2} & \sigma_{v02,03}\\ \sigma_{v01,03} & \sigma_{v02,03} & \sigma_{v03}^{3} \end{array}\right]$$

$$\left[\begin{array}{c} u_{01j}\\ u_{02j}\\ u_{03j}\\ u_{11j}\\ u_{12j}\\ u_{13j} \end{array}\right]∼MVN(0,\Omega_{u})=\left[\begin{array}{cccccc} \sigma_{u01}^{2} & \sigma_{u01,02} & \sigma_{u01,03} & \sigma_{u01,11} & \sigma_{u01,12} & \sigma_{u01,13}\\ \sigma_{u01,02} & \sigma_{u02}^{2} & \sigma_{u02,03} & \sigma_{u02,11} & \sigma_{u02,12} & \sigma_{u02,13}\\ \sigma_{u01,03} & \sigma_{u02,03} & \sigma_{u03}^{2} & \sigma_{u03,11} & \sigma_{u03,12} & \sigma_{u03,13}\\ \sigma_{u01,11} & \sigma_{u02,11} & \sigma_{u03,11} & \sigma_{u11}^{2} & \sigma_{u11,12} & \sigma_{u11,13}\\ \sigma_{u01,12} & \sigma_{u02,12} & \sigma_{u03,12} & \sigma_{u11,12} & \sigma_{u12}^{2} & \sigma_{u12,13}\\ \sigma_{u01,13} & \sigma_{u02,13} & \sigma_{u03,13} & \sigma_{u11,13} & \sigma_{u12,13} & \sigma_{u13}^{2} \end{array}\right]$$

$$\left[\begin{array}{c} e_{01ijk}\\ e_{02ijk}\\ e_{03ijk} \end{array}\right]∼MVN(0,\Omega_{\text{e}}):\Omega_{\text{e}}=\left[\begin{array}{ccc} \sigma_{e01}^{2} & \sigma_{e01,02} & \sigma_{e01,03}\\ \sigma_{e01,02} & \sigma_{e02}^{2} & \sigma_{e02,03}\\ \sigma_{e01,03} & \sigma_{e02,03} & \sigma_{e03}^{2} \end{array}\right]$$

*y*_*ijk*_ is a threefold set of outcome variables defined by the dummy variables *z*_1*ijk*_ (concentration problems), *z*_2*ijk*_ (prosocial behavior) and *z*_3*ijk*_ (disruptive behavior). The data set has a long format with three observations per case, which adds an artificial level to fit three equations simultaneously. The subscripts “i”, “j” and “k” denote the levels of occasions (time), children and schools, respectively. *β*_01_, *β*_02_, and *β*_03_ correspond to the intercepts of each measure, which are allowed to vary randomly at the levels of children and schools. *β*_11*j*_, *β*_12*j*_ and *β*_13*j*_ are the growth rates for each of the outcome measures, which are allowed to vary randomly across children. The growth rates have the associated errors denoted by *u*_11*j*_, *u*_12*j*_ and *u*_13*j*_. Time is treated flexibly via the addition of fixed squared and cubic terms for time, whose effects are denoted by the set of coefficients *β*_21_, *β*_22_, *β*_23_, *β*_31_, *β*_32_ and *β*_33_. The fixed part of the model also includes a set of coefficients *β*_41_, *β*_42_ and *β*_43_, which correspond to the main effect of the GBG trial on each of the outcomes. The growth rates for each outcome are also allowed to differ between the arms of the trial, by specifying a cross-level interaction effect between GBG and time (and its polynomials), whose coefficients are denoted by *β*_51_, *β*_52_ and *β*_53_ (polynomials: *β*_61_, *β*_62_, *β*_63_, *β*_71_, *β*_72_ and *β*_73_). A further set of covariates (see Table 1) are specified in the full model but are omitted here for simplicity. The random part of the model is split into three variance-covariance matrices Ω_*v*_, Ω_*u*_ and Ω_*e*_, which correspond to the levels of schools, children and occasions, respectively. Each diagonal element of matrices Ω_*v*_ and Ω_*e*_ correspond to the variances of the intercepts of the three outcomes at the school and occasion level, while the off-diagonal elements are the covariances between them. In matrix Ω_*u*_, the first three diagonal elements are the intercepts for the outcomes and the last three are the variances of the slopes of the linear terms for time (growth rates), while its off-diagonal elements correspond to the covariances between the intercepts and the slopes.

## Appendix B Exemplar code to fit the models

This is an example of how the models presented here can be fitted in the R package “R2MLwiN”. The model in Appendix A would be fitted as follows:

“Model_A” is a multivariate multilevel unconditional linear growth curve model fitted via the default settings of the MCMC algorithm as implemented in MLwiN. As mentioned before, the model in Appendix A has been kept deliberately simpler than the actual full model to improve readability. The full model presented in this article is fitted as follows:

This code presupposes the installation of R, MLwiN and the R package “R2MLwiN”. Indentation is not required. Categorical variables (“fsm”, “gbg”, “male” and “risk”) are coded as binary.

## Appendix C Multilevel model for missingness in the full model

We fitted a model to explore the factors associated with missingness in the full model. The outcome variable is a binary variable to indicate whether the observation (child) is missing from the full fitted model (Table 6). The covariates of the full model were used only as main effects. Table C.1 shows there is not enough evidence to support the hypothesis that any covariate is associated with missingness in the final model (confidence intervals overlap with zero). A caveat to these results is that the at-risk indicator is missing for 114 out of 147 children who were missing from the final model. A full information maximum likelihood (FIML) specification for the missingness model was attempted, but led to estimation issues, hence the model presented in Table C.1 is for complete cases of the at-risk indicator. All other observations are included (*N* = 2970; 96.3% of the full sample). Given the above, the data are unlikely to be missing completely at random (MCAR) and hence we assume them to be missing at random (MAR).

## References

[bb0005] Barrish H.H., Saunders M., Wolf M.M. (1969). Good behavior game: Effects of individual contingencies for group consequences on disruptive behavior in a classroom. Journal of Applied Behavior Analysis.

[bb0010] Best J.R., Miller P.H., Jones L.L. (2009). Executive functions after age 5: Changes and correlates. Developmental Review.

[bb0015] Bradshaw C.P., Waasdorp T.E., Leaf P.J. (2012). Effects of school-wide positive behavioral interventions and supports on child behavior problems. Pediatrics.

[bb0020] Breslau N., Breslau J., Peterson E., Miller E., Lucia V.C., Bohnert K., Nigg J. (2010). Change in teachers’ ratings of attention problems and subsequent change in academic achievement: A prospective analysis. Psychological Medicine.

[bb0025] Bryk A., Raudenbush S. (1987). Application of hierarchical linear models to assessing change. Psychological Bulletin.

[bb0030] Chan G., Foxcroft D., Smurthwaite B., Coombes L., Allen D. (2012).

[bb0035] D’Amico F., Knapp M., Beecham J., Sandberg S., Taylor E., Sayal K. (2014). Use of services and associated costs for young adults with childhood hyperactivity/conduct problems: 20-year follow-up. The British Journal of Psychiatry: the Journal of Mental Science.

[bb0040] Department for Education (2012). Pupil behaviour in schools in England. https://assets.publishing.service.gov.uk/government/uploads/system/uploads/attachment_data/file/184078/DFE-RR218.pdf.

[bb0045] Department for Work and Pensions (2013). Free school meal entitlement and child poverty in England. https://www.gov.uk/government/statistics/free-school-meal-entitlement-and-child-poverty-in-england.

[bb0050] Dion E., Roux C., Landry D., Fuchs D., Wehby J., Dupéré V. (2011). Improving attention and preventing reading difficulties among low-income first-graders: A randomized study. Prevention Science.

[bb0055] Dolan L.J., Kellam S.G., Brown C.H., Werthamer-Larsson L., Rebok G.W., Mayer L.S., Laudolff J., Turkkan J.S., Ford C., Wheeler L. (1993). The short-term impact of two classroom-based preventive interventions on aggressive and shy behaviors and poor achievement. Journal of Applied Developmental Psychology.

[bb0060] Donaldson J.M., Wiskow K.M., Teasdale B., Bradley M.S. (2017). Preventing crime and violence.

[bb0065] van Doorn J., van den Bergh D., Böhm U., Dablander F., Derks K., Draws T., Etz A., Evans N.J., Gronau Q.F., Haaf J.M., Hinne M., Kucharský Š., Ly A., Marsman M., Matzke D., Gupta A.R.K.N., Sarafoglou A., Stefan A., Voelkel J.G., Wagenmakers E.J. (2020). The JASP guidelines for conducting and reporting a Bayesian analysis. Psychonomic Bulletin and Review.

[bb0070] Durlak J.A. (2015). Studying program implementation is not easy but it is essential. Prevention Science.

[bb0075] Durlak J.A., Weissberg R.P., Dymnicki A.B., Taylor R.D., Schellinger K.B. (2011). The impact of enhancing students’ social and emotional learning: A meta-analysis of school-based universal interventions. Child Development.

[bb0080] Ford C., Keegan N., Poduska J., Kellam S., Littman J. (2014).

[bb0085] Gneezy U., Leonard K.L., List J.A. (2009). Gender differences in competition: Evidence from a matrilineal and a patriarchal society. Econometrica.

[bb0090] Goldstein H. (1987).

[bb0095] Goldstein H., Bock R. (1989). Multilevel analysis of educational data.

[bb0100] Goldstein H. (2011).

[bb0105] Goodman R. (1997). The strengths and difficulties questionnaire: A research note. Journal of Child Psychology and Psychiatry.

[bb0110] Greenberg M.T., Abenavoli R. (2017). Universal interventions: Fully exploring their impacts and potential to produce population-level impacts. Journal of Research on Educational Effectiveness.

[bb0115] Grimm K., Ram N., Estabrook R. (2017).

[bb0120] Gupta S.K. (2011). Intention-to-treat concept: A review. Perspectives in Clinical Research.

[bb0125] Hallquist M.N., Wiley J.F. (2018). MplusAutomation: An R package for facilitating large-scale latent variable analyses in Mplus. Structural Equation Modeling.

[bb0130] Hansen W.B., Bishop D.C., Jackson-Newsom J. (2010). Impact of a classroom behavior management intervention on teacher risk ratings for student behavior. Journal of Drug Education.

[bb0135] Hill K.G., Woodward D., Woelfel T., Hawkins J.D., Green S. (2016). Planning for long-term follow-up: Strategies learned from longitudinal studies. Prevention Science.

[bb0140] Hox J., Moerbeek M., van de Schoot R. (2017).

[bb0145] Hu L., Bentler P. (1999). Cutoff criteria for fit indexes in covariance structure analysis: Conventional criteria versus new alternatives. Structural Equation Modeling: A Multidisciplinary Journal.

[bb0150] Hubbard S., Masyn K.E., Poduska J., Schaeffer C.M., Petras H., Ialongo N., Kellam S. (2006). A comparison of girls’ and boys’ aggressive-disruptive behavior trajectories across elementary school: Prediction to young adult antisocial outcomes. Journal of Consulting and Clinical Psychology.

[bb0155] Humphrey N., Hennessey A., Ashworth E., Frearson K., Black L., Petersen K., Wo L., Panayiotou M., Lendrum A., Wigelsworth M., Birchinall L., Squires G., Pampaka M. (2018). https://educationendowmentfoundation.org.uk/public/files/GBG_evaluation_report.pdf.

[bb0160] Humphrey N., Hennessey A., Troncoso P., Panayiotou M., Black L., Peterson K., Lendrum A. (2021).

[bb0165] Ialongo N.S., Domitrovich C., Embry D., Greenberg M., Lawson A., Becker K.D., Bradshaw C. (2019). A randomized controlled trial of the combination of two school-based universal preventive interventions. Developmental Psychology.

[bb0170] Ialongo N.S., Werthamer L., Kellam S.G., Brown C.H., Wang S., Lin Y. (1999). Proximal impact of two first-grade preventive interventions on the early risk behaviors for later substance abuse, depression, and antisocial behavior. American Journal of Community Psychology.

[bb0175] Jiang D., Santos R., Josephson W., Mayer T., Boyd L. (2018). A comparison of variable- and person-oriented approaches in evaluating a universal preventive intervention. Prevention Science.

[bb0180] Kellam S.G., Mackenzie A.C.L., Brown C.H., Poduska J.M., Wang W., Petras H., Wilcox H.C. (2011). The good behavior game and the future of prevention and treatment. Addiction Science & Clinical Practice.

[bb0185] Kellam S.G., Rebok G.W., Ialongo N., Mayer L.S. (1994). The course and malleability of aggressive behavior from early first grade into middle school: Results of a developmental epidemiologically-based preventive trial. Journal of Child Psychology and Psychiatry, and Allied Disciplines.

[bb0190] Knapp M., King D., Healey A., Thomas C. (2011). Economic outcomes in adulthood and their associations with antisocial conduct, attention deficit and anxiety problems in childhood. The Journal of Mental Health Policy and Economics.

[bb0195] Korpershoek H., Harms T., de Boer H., van Kuijk M., Doolaard S. (2016). A meta-analysis of the effects of classroom management strategies and classroom management programs on students’ academic, behavioral, emotional, and motivational outcomes. Review of Educational Research.

[bb0200] Koth C.W., Bradshaw C.P., Leaf P.J. (2009). Teacher Observation of Classroom Adaptation-Checklist: Development and factor structure. Measurement and Evaluation in Counseling and Development.

[bb0205] Kourkounasiou M.A., Skordilis E.K. (2014). Validity and reliability evidence of the TOCA–C in a sample of Greek students. Psychological Reports.

[bb0210] Leflot G., Van Lier P., Onghena P., Colpin H. (2010). The role of teacher behavior management in the development of disruptive behaviors: An intervention study with the good behavior game. Journal of Abnormal Child Psychology.

[bb0215] van Lier P.A.C., Muthén B.O., van der Sar R.M., Crijnen A.M. (2004). Preventing disruptive behavior in elementary schoolchildren: Impact of a universal classroom-based intervention. Journal of Consulting and Clinical Psychology.

[bb0220] Moerbeek M. (2019). Bayesian evaluation of informative hypotheses in cluster-randomized trials. Behavior Research Methods.

[bb0225] Moffitt T.E., Scott S. (2008). Rutter’s child and adolescent psychiatry.

[bb0230] Muthén L., Muthén B. (2017).

[bb0235] Newsom J. (2015).

[bb0240] Nix R., Bierman K.L., Heinrichs B.S., Gest S.D., Welsh J., Domitrovich C.E. (2016). The randomized-controlled trial of Head Start REDI: Sustained effects on developmental trajectories of social-emotional functioning. Journal of Consulting and Clinical Psychology.

[bb0245] O'Keeffe J. (2019). A feasibility study and a pilot cluster randomised controlled trial of the PAX ‘Good Behaviour Game’ in disadvantaged schools. https://pureadmin.qub.ac.uk/ws/portalfiles/portal/185361886/PAX_GBG_PhD_2019.pdf.

[bb0250] Plewis I. (1996). Statistical methods for understanding cognitive growth: A review, a synthesis and an application. British Journal of Mathematical and Statistical Psychology.

[bb0255] Plewis I. (2005). Modelling behaviour with multivariate multilevel growth curves. Methodology.

[bb0260] Plewis I., Peterson P., Baker P.E., McGaw B. (2010). International encyclopedia of education.

[bb0265] R Core Team (2019). http://www.r-project.org.

[bb0270] Rabiner D.L., Godwin J., Dodge K.A. (2016). Predicting academic achievement and attainment: The contribution of early academic skills, attention difficulties, and social competence. School Psychology Review.

[bb0275] Rasbash J., Charlton C., Browne W., Healy M., Cameron B. (2020).

[bb0280] Reid J.B., Eddy J.M., Fetrow R.A., Stoolmiller M. (1999). Description and immediate impacts of a preventive intervention for conduct problems. American Journal of Community Psychology.

[bb0285] Reupert A., Woodcock S. (2010). Success and near misses: Pre-service teachers’ use, confidence and success in various classroom management strategies. Teaching and Teacher Education.

[bb0290] Rosseel Y. (2012). lavaan: An R package for structural equation modeling. Journal of Statistical Software.

[bb0295] Smith E.P., Osgood D.W., Oh Y., Caldwell L.C. (2018). Promoting afterschool quality and positive youth development: Cluster randomized trial of the Pax Good Behavior Game. Prevention Science.

[bb0300] Smith S., Barajas K., Ellis B., Moore C., McCauley S., Reichow B. (2021). A meta-analytic review of randomized controlled trials of the Good Behavior Game. Behavior Modification.

[bb0305] Spiegelhalter D., Best N., Carlin B., van der Linde A. (2002). Bayesian measures of model complexity and fit. Journal of the Royal Statistical Society, Series B (Statistical Methodology).

[bb0310] Streimann K., Selart A., Trummal A. (2020). Effectiveness of a universal, classroom-based preventive intervention (PAX GBG) in Estonia: A cluster-randomized controlled trial. Prevention Science.

[bb0315] Tanner-Smith E.E., Durlak J.A., Marx R.A. (2018). Empirically based mean effect size distributions for universal prevention programs targeting school-aged youth: A review of meta-analyses. Prevention Science.

[bb0320] Tolan P., Elreda L.M., Bradshaw C.P., Downer J.T., Ialongo N. (2020). Randomized trial testing the integration of the Good Behavior Game and MyTeachingPartnerTM: The moderating role of distress among new teachers on student outcomes. Journal of School Psychology.

[bb0325] Troncoso P. (2019). A two-fold indicator of school performance and the cost of ignoring it. International Journal of Educational Research.

[bb0330] Van der Leeden R. (1998). Multilevel analysis of repeated measures data. Quality and Quantity.

[bb0335] Witvliet M., van Lier P.A.C., Cuijpers P., Koot H.M. (2009). Testing links between childhood positive peer relations and externalizing outcomes through a randomized controlled intervention study. Journal of Consulting and Clinical Psychology.

[bb0340] Zhang Z., Parker R., Charlton C., Leckie G., Browne W. (2016). R2MLwiN: A package to run MLwiN from within R. Journal of Statistical Software.

